# Evidence review of hydroxyurea for the prevention of sickle cell complications in low-income countries

**DOI:** 10.1136/archdischild-2012-302387

**Published:** 2013-08-30

**Authors:** Mercy Mulaku, Newton Opiyo, Jamlick Karumbi, Grace Kitonyi, Grace Thoithi, Mike English

**Affiliations:** 1 School of Pharmacy, University of Nairobi, Nairobi, Kenya; 2 SIRCLE Collaboration, KEMRI-Wellcome Trust Research Programme, Nairobi, Kenya; 3 KEMRI-Wellcome Trust Research Programme; 4 Hematology and Blood Transfusion Unit, School of Medicine, University of Nairobi, Nairobi, Kenya; 5 Nuffield Department of Medicine, University of Oxford, Oxford, UK

**Keywords:** Genetics, Haematology

## Abstract

Hydroxyurea is widely used in high-income countries for the management of sickle cell disease (SCD) in children. In Kenyan clinical guidelines, hydroxyurea is only recommended for adults with SCD. Yet many deaths from SCD occur in early childhood, deaths that might be prevented by an effective, disease modifying intervention. The aim of this review was to summarise the available evidence on the efficacy, effectiveness and safety of hydroxyurea in the management of SCD in children below 5 years of age to support guideline development in Kenya. We undertook a systematic review and used the Grading of Recommendations Assessment, Development and Evaluation system to appraise the quality of identified evidence. Overall, available evidence from 1 systematic review (n=26 studies), 2 randomised controlled trials (n=354 children), 14 observational studies and 2 National Institute of Health reports suggest that hydroxyurea may be associated with improved fetal haemoglobin levels, reduced rates of hospitalisation, reduced episodes of acute chest syndrome and decreased frequency of pain events in children with SCD. However, it is associated with adverse events (eg, neutropenia) when high to maximum tolerated doses are used. Evidence is lacking on whether hydroxyurea improves survival if given to young children. Majority of the included studies were of low quality and mainly from high-income countries. Overall, available limited evidence suggests that hydroxyurea may improve morbidity and haematological outcomes in SCD in children aged below 5 years and appears safe in settings able to provide consistent haematological monitoring.

**What is already known on this topic?**

- Hydroxyurea represents the only widely used drug which modifies sickle cell disease (SCD) pathogenesis.
- In adults hydroxyurea has transformed the treatment of SCD and is associated with decreases in hospitalisations, acute chest syndrome episodes and painful crises and improved quality of life.

**What this study adds**

- Evidence on potential benefits of use of hydroxyurea on children below 5 years with SCD is that it is associated with decreased pain crises and dactylitis.
- Contextual issues of a low-income country like Kenya that may influence recommendation for use of hydroxyurea in children with SCD.

## Introduction

It is estimated that 7% of the world's population are carriers for haemoglobin disorders (sickle cell anaemia (SCA) and thalassaemia) and between 300 000 and 500 000 infants with the severe heterozygous forms of these diseases are born each year.^1^ Sickle cell disease (SCD) is the most important potentially devastating, recessively inherited condition. The well-described protective effect of sickle cell trait on mortality from malaria, and consequentially from serious bacterial diseases,^2^ has resulted in high prevalence of this gene disorder in many African countries. In SCD, deformation of red blood cells (‘sickling’) and vaso-occlusive phenomena are characteristic and result in pain, tissue injury and haemolysis.^3^ Disease severity varies widely but overall mortality is substantially increased and life expectancy decreased when compared with the general population. Despite the availability of effective treatments for improving clinical outcomes of SCD, mortality is high in children aged between 6 months and 3 years in Africa,^4^ ^5^ often before confirmatory diagnosis in such settings. Early detection and intervention may reduce this high burden of disease.

Treatment of patients with SCD is largely supportive with hydroxyurea (HU) representing the only widely used drug which modifies disease pathogenesis. It improves clinical outcomes by increasing fetal haemoglobin (HbF), which in turn reduces risks of ‘sickling’ events. In adults, it is associated with decreases in hospitalisations, acute chest syndrome episodes and painful crises, and improved quality of life.^6^ It was approved by the US Food and Drug Administration for treatment of SCD in adults in 1998 and has also become widely used for management of children with SCD in high-income countries.^7^

In Kenya, current clinical guidelines recommend HU only for adults who present with more than three sickle cell crises in a year. For children only supportive care (analgesics, supplementary folic acid and malaria prophylaxis when travelling to malaria endemic zones, penicillin prophylaxis and blood transfusion whenever necessary) is necessary.^8^ To help update clinical recommendations for care of children below 5 years of age with SCD in Kenya, we conducted a review of the available evidence on the effectiveness and safety of HU, compared with standard supportive care, on SCD-related mortality and morbidity. As making practice recommendations requires consideration of the quality of research evidence and local context, we also highlight contextual issues likely to be of importance to Kenya and countries with similar health systems challenges.

## Methods

### Search strategy

A standard search was performed in the Cochrane Library, MEDLINE (using the PubMed clinical query filters),^9^ and one clinical trials registry (http://clinicaltrials.gov). Reference lists of identified relevant articles were scanned to identify additional studies for inclusion. The search terms used were: (child OR neonate OR newborn OR infant) AND (SCD OR SCA) AND (HU OR hydroxycarbamide). No language limits were applied. Having identified a well-conducted systematic review published in December 2008,^7^ we restricted our search to studies published between January 2007 and March 2012 to supplement the findings of the existing review. Our purpose was not, however, to formally update this systematic review.

### Study selection criteria

Studies with more than 10 participants, randomised controlled trials (RCTs) and observational studies, in low-income and high-income countries were eligible for inclusion. Case reports, letters and commentaries were excluded. We also considered two National Institute of Health (NIH) reports on HU treatment for SCD.^10^ ^11^ Although our focus was on children aged below 5 years, we also considered relevant studies which enrolled children up to 18 years of age as there is a paucity of data on younger children. The outcomes specified as ‘critical’ included: mortality, rates of hospitalisation and severe neurological events (cerebrovascular accident). Outcomes considered important were: morbidity (ie, pain episodes) and toxicity (see table 1). We also extracted data on additional relevant outcomes reported in the identified studies including treatment effect on HbF level and organ function (see tables 2 and 3). Three reviewers independently screened titles, abstracts and full articles, and applied the predefined study selection criteria to identify eligible studies.

**Table 1:** Summary outcomes for children receiving hydroxyurea for sickle cell disease

Outcome	Effect
Blood markers	
Haemoglobin level	Not significantly different
Percentage of fetal haemoglobin	Increased (very low quality evidence)
Clinical outcomes	
Pain crises	Decreased (low quality evidence)
Hospitalisations	Decreased (low quality evidence)
Blood transfusion therapy	Insufficient data
Acute chest syndrome	Insufficient data
Secondary stroke	Decreased (very low quality evidence) No effect (low quality evidence)
Prevention of end organ damage	
Spleen	No significant difference (low quality evidence)
Kidney	No significant difference (low quality evidence)
Brain (transcranial Doppler velocity)	Decreased (very low quality evidence)
Mortality	
Mortality	Decreased effect
Toxicity	
Neutropenia	Mild to moderate (moderate quality evidence)
Leg ulcers	Insufficient data
Thrombocytopenia	Insufficient data
Anaemia	Insufficient data

**Table 2:** Grade summary of findings for Wang *et al* 2011^19^

Outcomes	Illustrative comparative risks* (95% CI)		Relative effect (95% CI)	No. of participants (studies)	Quality of the evidence (GRADE)
	Assumed risk	Corresponding risk			
	Placebo	Hydroxyurea			
Spleen function Spleen scan uptake	Moderate		Mean difference −11% (−26 to 5)	144 (1 study)	⊕⊕⊝⊝ low†‡§
Renal function Mean DTPA GFR mL/min per 1.73 m^2^	The mean renal function in the control groups was mL/min per 1.73 m^2^	The mean renal function in the intervention groups was 2 higher (16 lower to 20 higher)	Mean difference 2 mL/min per 1.73 m^2^ (−16 to 20)	133 (1 study)	⊕⊕⊝⊝ low†§
Haematological data(HbF) Percentage	The mean haematological data at exit in the control groups was 17.1%	The mean haematological data at exit in the intervention groups was 22.4% (5.3% higher)	Mean difference 6.7 (4.8 to 8.7)	158 (1 study)	⊕⊕⊝⊝ low†§
Pain episodes (vaso-occlusive pain episodes) Number of events	Study population		HR 0.59 (0.42 to 0.83)	193 (1 study)	⊕⊕⊝⊝ low†
	773 per 1000	583 per 1000 (464 to 708)			
	Moderate				
Number of transfusions Number of events	Study population		HR 0.55 (0.32 to 0.96)	193 (1 study)	⊕⊕⊝⊝ low†
	340 per 1000	204 per 1000 (125 to 329)			
	Moderate				
Acute chest syndrome Number of events	Study population		HR 0.36 (0.15 to 0.87)	193 (1 study)	⊕⊕⊝⊝ low†§
	186 per 1000	71 per 1000 (30 to 164)			
	Moderate				
Dactylitis Number of events	Study population		HR 0.27 (0.15 to 0.5)	193 (1 study)	⊕⊕⊝⊝ low†§
	433 per 1000	142 per 1000 (82 to 247)			
	Moderate				
Rate of hospitalisation Number of events	Study population		HR 0.73 (0.53 to 1)	193 (1 study)	⊕⊕⊝⊝ low†§
	866 per 1000	769 per 1000 (655 to 866)			
	Moderate				
Moderate neutropenia Absolute neutrophil count Follow-up: 24 months	Study population		HR 3.0 (1.7 to 5.1)	193 (1 study)	⊕⊕⊝⊝ low†§
	186 per 1000 Moderate High	460 per 1000 (295 to 649)			

*The basis for the assumed risk (eg, the median control group risk across studies) is provided in footnotes. The corresponding risk (and its 95% CI) is based on the assumed risk in the comparison group and the relative effect of the intervention (and its 95% CI).

†Trial downgraded due to indirectness (trial done in USA).

‡Wide CIs.

§Small sample size/small number of events (decreased spleen function events, 19/70 in the HU group and 28/74 in the placebo group).

GRADE Working Group grades of evidence; High quality: Further research is very unlikely to change our confidence in the estimate of effect. Moderate quality: Further research is likely to have an important impact on our confidence in the estimate of effect and may change the estimate. Low quality: Further research is very likely to have an important impact on our confidence in the estimate of effect and is likely to change the estimate. Very low quality: We are very uncertain about the estimate.

ANC, absolute neutrophil count; DTPA, diethylenetriaminepentaacetic acid; GFR, glomerular filtration rate; GRADE, Grading of Recommendations Assessment, Development and Evaluation; HbF, fetal haemoglobin; HU, hydroxyurea; RR, rate ratio.

**Table 3:** Grade summary of findings tables for the observational studies^12–17^

Outcomes	Illustrative comparative risks* (95% CI)		Relative effect (95% CI)	No. of participants (studies)	Quality of the evidence (GRADE)
	Assumed risk	Corresponding risk			
		Hydroxyurea			
Haematological data (HbF) Percentage	Not estimable	Not estimable	Significant increase in HbF after HU use In one study the change was 11.2 (7.1 to 15.4).^13^ In another HbF changed from 19.8±6.9 to 24.4±6.3^17^	45 (3 studies)	⊕⊝⊝⊝ very low†	
Pain episodes (vaso occlusive pain events) Number of events	Study population		RR 0.79 (0.71 to 0.89)	523 (1 study)	⊕⊝⊝⊝ very low‡
	Moderate				
Hospitalisation Number of events	Study population		HR 0.65 (0.43 to 0.97)	312 (1 study)	⊕⊝⊝⊝ very low§¶
	Moderate				
HRQL PedsQL Score	The mean HRQL using pedsQL self report in the control groups was 69 scale scores	The mean HRQL using pedsQL self report in the intervention groups was 75 higher (62 to 86.4 higher)**	HU group=75 (62.0 to 86.4) No HU group=69 (54.1 to 79.9)	191 (1 study)	⊕⊝⊝⊝ very low**,††,‡‡
Neurological event number of events Follow-up: 111 patient-years	Study population		HR 9.4 (1.3 to 70.6)	43 (1 study)	⊕⊝⊝⊝ very low§§
	606 per 1000	1000 per 1000 (702 to 1000)			
	Moderate				

*The basis for the assumed risk (eg, the median control group risk across studies) is provided in footnotes. The corresponding risk (and its 95% CI) is based on the assumed risk in the comparison group and the relative effect of the intervention (and its 95% CI).

†There were no comparison groups, the studies were before and after studies.

‡Patient assignment to groups incompletely reported.

§Before and after design.

¶Downgraded due to indirectness (study done in North Carolina).

**Small sample size (n=191) leading to wide CI (imprecision).

††Selection bias likely as patient selection to comparison study groups done by investigator.

‡‡Downgraded due to indirectness (study conducted in North America).

§§Small sample size (n=43) leading to wide CI (imprecision).

GRADE Working Group grades of evidence; High quality: Further research is very unlikely to change our confidence in the estimate of effect. Moderate quality: Further research is likely to have an important impact on our confidence in the estimate of effect and may change the estimate. Low quality: Further research is very likely to have an important impact on our confidence in the estimate of effect and is likely to change the estimate. Very low quality: We are very uncertain about the estimate.

GRADE, Grading of Recommendations Assessment, Development and Evaluation; HbF, fetal haemoglobin; HRQL, health related quality of life; HU, hydroxyurea; PedsQL, paediatric quality of life; RR, rate ratio.

### Data extraction

Data were abstracted by a single reviewer and a co-investigator verified accuracy. Abstracted data included: study characteristics (eg, study design, settings), doses of HU, duration of HU therapy and outcome measures (see web only table 5).

### Data synthesis

There was heterogeneity due to differences in study designs (RCTs vs observational studies) and outcome measures, so statistical pooling of results was considered inappropriate. The results are therefore presented as a narrative summary.

### Quality assessment

Assessment of the quality of evidence was done using the Grading of Recommendations Assessment, Development and Evaluation tool.^18^ This was done by two reviewers independently and disagreements resolved through discussion. The unique features of Grading of Recommendations Assessment, Development and Evaluation include: (1) explicit, comprehensive criteria for downgrading and upgrading quality of evidence ratings; (2) explicit evaluation of the importance of outcomes; and (3) clear separation of quality of evidence from the strength of care recommendations. The approach classifies the quality of evidence into four categories: high, moderate, low or very low. The quality of evidence is taken into account in the narrative synthesis of findings.

## Results

**Figure 1:**
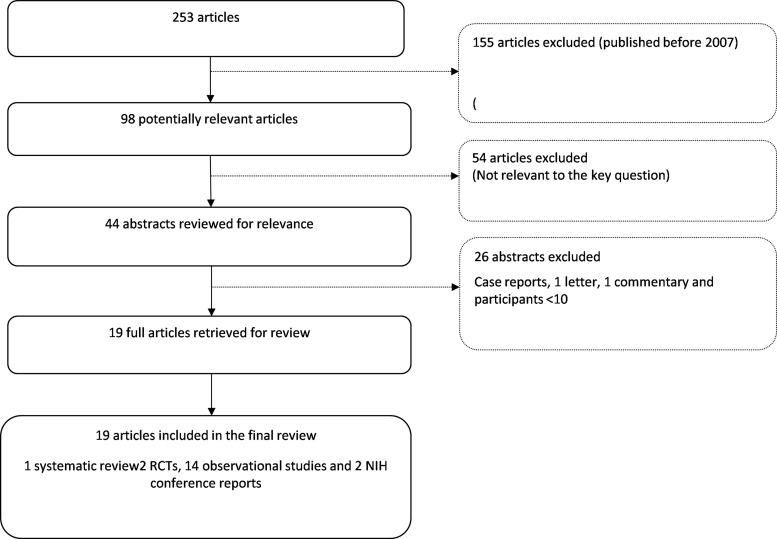
Flow diagram of the study selection criteria.

Overall, we identified 98 studies from the searches and included 19 reports in this review (1 systematic review,^7^ 2 multicentre RCTs^19^ ^20^ and 16 observational studies inclusive of two NIH reports^10–16^ ^21–28^ (figure 1)). The majority of included studies were conducted in high-income countries (n=11); only two studies were conducted in low-income and middle-income countries. The doses of HU were varied (range: 10 mg/kg/day to 35 mg/kg/day). Control treatments were placebo (in one RCT) and standard treatment without HU.

### A. Systematic review

The 2008 systematic review^7^ included 1 RCT (n=25) and 22 observational studies (that included 15–225 participants and 3 case reports). Children studied ranged from ages 2–22 years. The authors concluded that there was high quality evidence suggesting that use of HU resulted in: reductions in hospitalisation events (n=5 studies, 56% to 87% decline in yearly rate); and increased total haemoglobin (n=16 studies, 5% to 20%) and HbF (n=17 studies, 93% to 366% increase) in children with severe SCA. They also stated that there was moderate quality evidence suggesting that HU reduced painful crisis (n=5 studies). In addition, low quality evidence suggested that HU was associated with decreased transfusions (three studies) and neurological events (three studies), and improvement in splenic function (three studies). Common adverse events were of mild to moderate severity and included: mild to moderate neutropenia (500 to <1500/µL)^3^, mild thrombocytopenia (<80×10^3^/µL)^3^, severe anaemia (haemoglobin <50 g/L), rash or nail changes and headache. Severe adverse events (eg, leukaemia or secondary malignancies) were rare and not clearly attributable to HU.

### B. Post systematic review RCTs and observational studies

One double-blind RCT^19^ (N=193) was conducted in 13 centres in the USA. It enrolled children aged 9–18 months with HbSS (homozygous type of SCA) or Hb S/ β-thalassaemia irrespective of clinical severity. HU was administered as a standard dose of 20 mg/kg without escalating to maximum tolerated dose. The primary end points were changes in spleen and renal function. There were no significant differences between the HU and the placebo groups in these end points: 19 of 70 (27%) patients had decreased spleen function at exit in the HU group versus 28 of 74 (38%) patients in the placebo group (mean difference −11%, 95% CI −26% to 5%, p=0.21) and a difference in the mean increase in the diethylenetriaminepentaacetic acid glomerular filtration rate in the HU group versus the placebo group of 2 mL/min per 1.73 m^2^, 95% CI −16 to 20, p=0.84. The quality of evidence for the reported outcomes was rated as low (table 2).

Data relevant to the critical and important outcomes that are the subject of this review are drawn from the two multicentre RCTs and 14 observational studies, and are summarised below.

### Mortality

Two studies reported on this outcome; in the RCT there were no deaths in the 24 months of follow-up.^19^ Nzouakou *et al*^22^ (n=123) reported four deaths in a retrospective cohort study during follow-up, though the patients had stopped taking HU 1–5 years before their deaths. The deaths were attributed to toxic shock, severe vaso-occlusive crises, heart failure and non-specified cardiac failure.

### Hospitalisations

There were no significant differences in hospitalisation rates between comparison groups in the RCT (HR=0.73, 95% CI 0.53 to 1.00, p=0.05).^19^ However, in one retrospective longitudinal study (n=312) adherence to HU resulted in decreased risk of SCD-related hospitalisations (HR=0.65, 95% CI 0.43 to 0.97, p=0.035),^12^ in the 1st year following initiation of HU. Nzouakou *et al*^22^ reported a mean decrease of 13.4 days of hospitalisation under HU in comparison with the period before HU initiation (p<0.0001) observed in 64 patients. Similarly in one prospective single-centre study (n=47) HU was associated with a decrease in mean hospitalisation days from 29.3 days/year (95% CI 7 to 84) before HU to 3.2 days/year (95% CI 0 to 15) after HU, p<0.01.^21^

### Neurological events

HU has been reported to prevent secondary stroke but not primary stroke in children. Ali *et al*^16^ (n=43), reported that only one child in the HU group had clinical stroke recurrence, incidence rate 2/100 person-years, compared with 20/33 in the non-HU group, incidence rate 29/100 person-years; HR 9.4, 95% CI 1.3 to 70.6, p=0.03. Four observational studies^13^ ^23–25^ reported data on other neurological end points: two^13^ ^25^ of them reported on transcranial Doppler (TCD) velocity (elevated velocities have been shown to be associated with increased risk of stroke)^25^. In one prospective pilot study (n=14),^13^ the average TCD values decreased with average reduction of 25.6±27.6 cm/s (95% CI 8.1 to 43.1, p<0.01) in the right middle cerebral artery (MCA) and 26.8±32.6 cm/s (95% CI 6.1 to 47.6, p<0.05) in the left MCA following use of HU. In the second study,^25^ HU resulted in a significant decrease in TCD velocity in the right MCA (166±27 cm/s to 135±27 cm/s, p<0.001) and the left MCA (168±26 cm/s to 142±27 cm/s, p<0.001). In one retrospective cohort study (n=52),^24^ 96% of the patients had stable MRI findings after HU while 28% of the study participants had MRI-identified silent brain ischaemia prior to use of HU. An additional non-inferiority randomised multicentre trial (n=161)^20^ compared standard treatment (transfusions/chelation, n=66) with alternative treatment (HU/phlebotomy, n=67) in children with SCA. The findings showed no benefit of HU on prevention of secondary stroke: there was no stroke on the transfusion/chelation arm but 7/67 (10%) on the HU/phlebotomy arm (which was still within non-inferiority stroke margin), and no difference in the liver iron concentration in comparison with the baseline in the two arms.

### Pain episodes

HU was associated with decreased frequency of pain events in four studies (one RCT and three observational studies). In the RCT,^19^ HU significantly reduced pain episodes over 24 months of follow-up (177 events in 62 patients in the HU group vs 375 events in 75 patients in the control group, p<0.002) compared with placebo. Similarly, HU was associated with significant reductions in pain episodes in the two retrospective studies and one prospective study: Stallworth *et al*^26^ (n=523), (Rate ratio, RR=0.79, 95% CI 0.71 to 0.89, p<0.0001); Candrilli *et al*^12^ (n=312), (HR=0.66, 95% CI 0.47 to 0.92, p=0.0130) and Mellouli *et al*^21^ (n=47), (21 out of 38 patients treated with HU for recurrent crises (>three crises/year) had no further crises).

### Fetal Haemoglobin (HbF)

Three studies (one RCT and two observational studies, n=213) reported statistically significant increases in HbF.^13^ ^14^ ^19^ In the RCT,^19^ HU was associated with a higher mean exit concentration of HbF (by 5.3%) compared with that in the placebo group (p<0.0001).

### Toxicity

Three studies^19^ ^21^ ^22^ reported on this outcome. The multicentre RCT reported that HU was associated with a significantly higher frequency of episodes of mild to moderate neutropenia that resulted in temporary treatment cessation (107 events in 45 patients vs 34 events in 18 patients in the placebo arm over 24 months; HR 3.0, 95% CI 1.7 to 4.1, p<0.001). Persistent or recurrent neutropenia (nine children in the HU arm vs five children in the placebo arm) led to a decrease in HU dosage to 17.5 mg/kg/day. One retrospective cohort study (without comparator data),^22^ (n=123) of children on HU treatment reported that 41 patients (33%) experienced 66 adverse events, including four deaths (described above), during a median follow-up of 2.8 years (range 0.02–10.5; frequency 12% per patient-year). The most frequent event in this study was leg ulcers; some had cutaneous reactions such as skin dryness and some minor haematological disorders. The 66 adverse events did not require changing HU dose for 25 (38%) episodes but led to dose reduction for 16 (24%) others and to HU withdrawal for 21 (32%). Mellouli *et al* (n=47) reported one episode of severe thrombocytopenia with severe leucopenia that resolved after stopping HU, another episode of pancytopenia was attributed to parvovirus infection.

Additional findings on adherence, health related quality of life outcomes and findings from the two NIH reports are summarised in web appendix 1.

## Discussion

### Interpretation of results

Adult studies are considered to provide strong evidence for the efficacy of HU with decreases in severe painful episodes, hospitalisations, number of blood transfusions and acute chest syndrome.^10^ The data also suggest that the risks of HU are acceptable in adults as compared with the risks of untreated SCD,^11^ with reduced mortality after 9 years of follow-up in those on treatment initiated for multiple vaso-occlusive crises.^6^

The available evidence on the efficacy, effectiveness and safety of HU in childhood SCD is mostly from high-income countries and for children up to 18 years of age. Studies on children below 5 years are very limited, with only one recent RCT enrolling children (n=193)^19^ from the age of 9–18 months, yet this age group experiences mortality rates as high as 7.3 per 100 person-years of observation in African settings, with children below 5 years accounting for 70% of all deaths.^29^ ^30^ Furthermore, mortality remains high in the group of children (aged >3 years) who may benefit from HU therapy if initiated early in life.^31^ Dosages of HU used in childhood studies ranged from 10 mg/kg/day to 35 mg/kg/day. In most studies, HU was started at the lower dosage and escalated to maximum tolerated dose with continuous haematological monitoring to safeguard against potential neutropenia.

A consistent feature of these studies was the provision of high quality supportive care in addition to HU. Of specific relevance to low-income settings is the use of regular haematological monitoring. Thus in the RCT conducted in early childhood full blood counts were drawn every 2–4 weeks except for HbF which was obtained every 6 months. Results were used to identify mild to moderate neutropenia and guide treatment cessation until blood counts normalised. The consequences of continuing HU therapy in the presence of mild to moderate neutropenia when haematological monitoring is either not possible at all or based on much reduced sampling frequency, is not known.

Given these caveats, available low quality evidence suggest that HU used in children, if adequately adhered to by patients and appropriately monitored, may be associated with reduced rates of hospitalisation and reduced frequency of painful vaso-occlusive crises. On the other hand low quality evidence suggests that HU is not beneficial in preventing secondary episodes of stroke^20^ (table 4). Data on longer term adverse events are very limited, few children under 5 years of age have been studied and follow-up times in the summarised studies was short.

**Table 4:** Grade summary of findings for Ware *et al* 2012^20^

Outcomes	Comparator arm Transfusions with chelation	Intervention arm Hydroxyurea with phlebotomy	Relative effect (95% CI)	No. of participants (studies)	Quality of the evidence (GRADE)	Comments
**Secondary stroke recurrence** recurrence rate Follow-up: 6 months	Recurrence rate of 0%, 0.0 events per 100 patient years in comparator arm versus recurrence rate of 10%, 5.6 events per 100 patient years in the intervention arm		Rate difference of 0.1	161 (1 study)	⊕⊕⊝⊝ low†‡§¶	
**Iron overload** Liver Iron concentration mg/g dry weight liver Follow-up: 6 months	The median iron overload in the control groups was 17.3 mg/g dry weight liver (8.8–30.7 IQR)	The median iron overload in the intervention groups was 17.2 mg/g dry weight liver (10.0–30.6 IQR)	Change from baseline(median (IQR) −2.2 (−5.5 to 4.9) in the comparator group −1.2 (−2.8 to 7.2) in the intervention group	161 (1 study)	⊕⊕⊝⊝ low †‡§¶,**,††	

*Children enrolled in the study were above 5 years old yet interested in data of children below 5 years.

†The use of hydroxyurea overlapped at some point with transfusions during the dose escalation.

‡The study setting is in high-income setting where there is thorough laboratory monitoring in contrast with low-income setting that the study question focuses on.

§Some point estimates in the study have wide CIs.

¶Sample sizes in both arms were small (hydroxyurea with phlebotomy, n=67; while transfusions with chelation, n=66).

GRADE Working Group grades of evidence; High quality: Further research is very unlikely to change our confidence in the estimate of effect. Moderate quality: Further research is likely to have an important impact on our confidence in the estimate of effect and may change the estimate. Low quality: Further research is very likely to have an important impact on our confidence in the estimate of effect and is likely to change the estimate. Very low quality: We are very uncertain about the estimate.

GRADE, Grading of Recommendations Assessment, Development and Evaluation.

### Weaknesses of the current literature

The majority of the studies summarised in the 2008 systematic review^7^ and published since this review, were observational (22 studies and 14 studies, respectively) increasing the possibility of bias and confounding. Further, most had retrospective designs with assignment to HU based on patient or physician preference and there was substantial heterogeneity in outcomes measured. Only one RCT enrolling children from infancy and early childhood has been published to date. Additional limitations of summarised evidence from a Kenyan perspective and when considering guidelines for those aged less than 5 years include: (1) indirectness of evidence: most studies were done in USA and enrolled children upto 18 years of age and adults; (2) very limited data on critical outcomes and; (3) imprecision as most studies had small numbers of participants (only two studies, both retrospective,^12^ ^26^ enrolled more than 300 patients).

### Implications for policy, practice and future research

Use of HU in children under 5 years of age with SCD who have vaso-occlusive crises and dactylitis and whose treatment can be appropriately monitored appears reasonable although the quality of evidence supporting this position is low to moderate.^32^ However, it is not yet clear if treatment as a prophylactic therapy can be recommended for children of this age with SCD, especially in low-income settings where the ability to monitor therapy may be limited.

Additional RCTs are needed to assess the effectiveness and safety of HU in children below 5 years in Africa ideally using pragmatic regimens with low frequency haematological monitoring. Further studies are also needed to establish the optimal dosing of HU. Such studies are justified as HU is relatively cheap. In Kenya, in the private sector, the average price for a 500 mg HU capsule is approximately US$0.47,^33^ which translates to treatment cost per month of about US$14. However, alternative formulations will be required for children and monitoring will increase treatment costs. Formal cost-effectiveness analyses to examine costs and health gains of HU are therefore needed to inform decisions on the feasibility and desirability of scaling up treatment in low-income settings such as Kenya.

## Conclusion

Our findings suggest that HU, the only available disease modifying therapy, might improve haematological outcomes (HbF), decrease hospitalisation rates and reduce pain episodes. Very low quality data exist on the effects of HU in children below 5 years in low-income countries where capacity for haematological monitoring is very limited. Further pragmatic RCTs with linked cost-effectiveness analyses are needed to address this gap.

## Supplementary Material

Web supplement
