# Supplementary material for: Evidence review of hydroxyurea for the prevention of sickle cell complications in low-income countries
Source: Arch Dis Child. 2013 Aug 30;98(11):908–14. doi: 10.1136/archdischild-2012-302387 (PMC3812872; doi:10.1136/archdischild-2012-302387)
Supplement: Web supplement [file archdischild-2012-302387-s1.pdf]

Web appendix 1. Adherence and health related quality of life outcomes

Adherence

Two studies reported on this outcome. Thornburg et al 2010 ,[21] a cross- sectional study (n= 75 children) reported an increase in Fetal Hemoglobin associated with good adherence measured with the parent/proxy Morisky score (mean change: 8.0%, 95% CI 6.2 to 9.8; p<0.0001). One retrospective longitudinal study, [14] (n=312) reported a 35% adherent rate to hydroxyurea defined as a medication possession ratio (MPR)≥0.80 (the mean MPR was 0.60). In the twelve months following hydroxyurea initiation, adherence was associated with decreased risk of SCD related hospitalization (HR=0.65, p=0.0351), decreased vaso-occlusive events (HR=0.66, p=0.0130), reduced costs (all cause and SCD related inpatient: $5,286, p<0.0001 and $4,403, p<0.0001 respectively; total costs, $6,529, p<0.0001 and $5,329; p<0.0001)

Health related Quality Of Life (HQRL)

In one retrospective cohort study, [22] (n=191) hydroxyurea was associated with a higher median (Inter quartile range, IQR) self reported peds quality of life score (pedsQL) compared to control (hydroxyurea group 75 (62.0, 86.4) versus control group 69.0 (54.1, 79.9); p=0.04).

National Institute of Health (NIH) reports on hydroxyurea treatment

The two NIH reports [10, 11] published in 2008 and based on systematic review data prepared through the Agency for Healthcare Research and Quality (AHRQ) and presentations by experts highlighted a number of issues related to the efficacy, effectiveness and safety of hydroxyurea, as briefly outlined below.

Evidence from the NIH reports;

Efficacy and effectiveness of hydroxyurea

The efficacy of hydroxyurea treatment for adults with SCD-SS (homologous genotype) is established. Although the evidence for efficacy of hydroxyurea treatment for children is not as strong, the emerging data encourages HU treatment in children with SCD on the basis of observational studies in both adults and children suggesting reductions in complications of SCD (including pain, hospitalizations, blood transfusions and the acute chest syndrome) and decreasing mortality.

Toxicity

Short term effects (within 6 months of HU initiation) included; decreased leukocyte count (leucopenia), decreased platelet count (thrombocytopenia), decreased erythrocyte count (anemia) and decreased reticulocyte count. Long term effects (more than 6months of HU initiation) include; birth defects in the offspring of people receiving the drug, growth delays in children receiving the drug and cancer in both children and adults who have received the drug. More information on the incidence and severity of these side effects was considered acceptable compared with the risks of untreated SCD in adults.

Web only Table 5: Characteristics of included studies

Author<br>Year	Design<br>Setting	Sample size<br>Population	Intervention	Comparator	Outcomes
**Wang et al 2011**	Multicentre randomized controlled trial<br><br>13 centres in USA	n=193<br><br>Age (9-18 months)<br>HbSS or Hb Thalassaemia	Hydroxyurea (20mg/kg)<br><br>96 children received it<br>1 incorrect diagnosis<br><br>Follow-up<br><br>4 withdrawals (3 lost to follow up, 1 incorrect diagnosis)<br><br>91 analyzed	Placebo<br><br>97 children<br><br>88 analyzed	**Primary endpoints**<br><br>1. Spleen function(splenic uptake on 99^{m} Tc-sulphur colloid liver spleen scans)<br><br>Decreased spleen function at exit (compared with baseline)<br>• Intervention: 19/70 (27% Failure)<br>• Comparator: 28/74 (38% Failure) (P value 0.21)<br><br>2. Renal function (mean DTPA GFR ml/min per 1.73 m^2)<br><br>Hydroxycarbamide group<br>(n=67), at entry Glomerular filtration Rate (GFR) was 123ml/min while at exit it was 146ml/min, having 18% difference<br><br>Placebo group<br>(n=66), at entry GFR was 125 ml/min while at exit 146ml/min, having a 17% difference. (P value 0.84, difference between Hydroxyurea group and placebo group)<br><br>**Secondary outcomes**<br>• HU decreased pain (177 events in 62 patients *vs.* 375 events in 75 patients in the placebo group, p=0.002<br>• Decreased dactylitis (24 events in 14 patients vs. 123 events in 75 patients in the placebo group<0.0001
**Thornburg et al 2010**	Single Institution Cross sectional Study,<br>Duke's university Medical Centre (DUMC).	n=75,-Age-<br>(<18yrs)<br>Children with SCA.	Hydroxyurea(mean dose 24mg/kg)	No comparator	Good adherence was estimated at 82% with visual analog scale,<br>84% with Morisky score,<br>85% with medical provider report<br>77% with clinic visits<br>49% on the basis of pharmacy refills.

					Increase in HbF was moderately associated with good adherence as measured with the parent/proxy Morisky<br>Score<br>R=-0.39,95% CI,-0.58—0.17;p<0.01<br>Prescription refills<br>R=0.39;95% CI 0.16-0.57;p<0.01
**Thornburg et al 2011**	Retrospective Cohort study,<br>Milwaukee in North America and DUMC	n=191,<br>Children with SCD<br>Age (2-18yrs)<br>Children on chronic transfusion therapy were excluded from the analysis because transfusions are disease modifying therapy.	Hydroxyurea<br>N=114	No hydroxyurea<br>N=77	**Primary outcome**<br>HRQL (Health related quality Of life) measured using<br>Median (Inter Quartile Range) Peds QL generic core scales.<br>children in HU group had higher median (IQR) PedsQL self report total scale scores than children in the no Hydroxyurea group [HU group 75 (62.0,86.4),no HU group 69.0 (54.1,79.9); p=0.04]<br><br>Child self-report physical functioning scores were significantly higher for children taking Hydroxyurea [HU group 79.7 (62.5,90.6), no HU group 71.4(58.6, 81.2); p=0.01]<br><br>Similarly, parent proxy-report physical functioning scores were significantly higher for children taking HU[Hydroxyurea group 75 (53.9,87.5),no HU group 71.9(53.2,90.6); p=0.05]
**Greenway et al 2011**	Single institution retrospective cohort study at DUMC	n=35-Children with SCD	Hydroxyurea/phlebotomy	Transfusions without Hydroxyurea	Stroke recurrence and other neurological outcomes.<br>At the end of extended follow up the average duration of transfusions was 7.2±6.1 years for those who continued Hydroxyurea therapy compared with 12.1±2.7 for those who returned to transfusion therapy(p=0.04)<br><br>The average lifetime number of transfusions was 84±98 in

					Those 20 patients compared with 523±256 for those 8 who restarted transfusion therapy ($p<0.001$).<br><br>During 14 year follow up period,10 of the original 35patients (29%) had a recurrent stroke while on hydroxyurea/Phlebotomy<br><br>The recurrent stroke event rate observed over the entire treatment and extended follow up period is 4.6 (95% CI 2.2-8.4) per 100 patient years.<br><br>4 of 20 (20%) patients who had transfusions overlapped with HU had recurrent stroke at median of 2.6 years (range 0.49-4.28 years) and 6 of 15(40%) patients who did not have transfusions overlapped with Hydroxyurea had recurrent stroke at a median of 0.94 years (range 0.19-7.08 years) ($p=0.006$)
**Thornburg et al 2009**	Prospective Pilot study (Follow up for two years) at Duke's Medical Centre.	n=14 Children with Sickle cell anemia<br>Age 18mnths-5yrs	Hydroxyurea(MTD 28mg/kg)	No comparator group(comparison of the values between entry and exit)	Assess safety and efficacy of HU<br>**Hematological Efficacy(n=14)**<br>HbF% Before 14.6±9.0, After 25.9±6.6), ($p<0.001$)<br>**Renal function (GFR)-n=11**<br>After 2 years, the average GFR value did not rise as expected in this age range(mean change 5.1ml/min/1.73m^2 ;95% CI=-4.39 to 14.6;p=0.26)<br>**Brain function (n=12)**<br>At study exit, average TCD values significantly decreased with an average reduction of 25.6±27.6 cm/sec in the right MCA (95% CI=8.1 to 43.1;$p<0.01$) and 26.8±32.6cm/sec in the left MCA(95% CI =6.1 to 47.6;$p<0.05$)<br>**Neurocognitive testing (n=9)**

					Between entry and exit, mean standard scores increased by 2.0 points, not statistically significant (95% CI=-21.4 to 25.5;p=0.70)<br>**HRQL Testing**<br>Mixed model analysis indicated no significant changes over time for global HRQL between study time points (slope=1.0;95% CI=-3.9 to 5.9;p=0.67)<br><br>However, IOF (Impact on Family) scores significantly decreased from baseline to exit (slope=-5.3;95% CI=-8.2 to -2.5;p=0.001),indicating parental Perception of less impact of the child's disease on family Functioning over time.
**Stallworth et al 2010**	Retrospective cohort study<br>From South Carolina Medicaid System.	n=523,Children with SCD(Age ≤17)<br>Control n =348<br>Intervention=<br>175 records	Hydroxyurea	Not treated with hydroxyurea	<u>**Those receiving care in specialized clinics.**</u><br>Pain Episodes (RR=0.79, p<0.0001)<br>Pain acute care (RR=0.90, p=0.01)<br><u>**Compared with the non-HU cohort, the HU group evinced**</u><br><u>**a significantly higher risk of experiencing vaso-occlusive**</u><br><u>**pain episodes**</u><br>Vaso-occlusive pain episodes (RR=3.32,p<0.0001) ACS/Pneumonia episodes (RR=2.66.p<0.0001), and higher outpatient, inpatient/emergency, and total service costs (RR=1.85, 2.11, 2.10 and p<0.0001 respectively) over time.
**Tripathi et al 2011**	Cohort study from South Carolina Medicaid Programme.	n=523,Children with SCD<br>(Age ≤17yrs)<br>(HbSS-homozygous)<br>Control N=348<br>Intervention<br>N=175	Hydroxyurea	Not treated with Hydroxyurea	<u>**Organic Specific complications**</u><br>More in HU treated group compared to non HU group<br>Cardiovascular complications[OR]=3.15, CI:1.97-5.03<br>Hepatic complications OR 5.41, CI 3.54-8.27<br>Renal complications OR 5.09; CI 3.37-7.67<br>Pulmonary complications OR 4.07; CI 1.88-8.79

**Hankins et al 2008**	Retrospective cohort study<br>9 year data collection	Children with SCD(3.0-17.6)<br>N=52<br>Median age 9.9 yrs	Hydroxyurea<br>Initial dose(15-20mg/kg/day)<br>MTD not exceed 30-35mg/kg/day	_	6/43 (non-splenectomized) -14% recovered splenic filtrative function.<br>• Brain<br>Before HU<br>7/25 (28%) had SBI (silent brain Ischemia)<br>17/25 (68%) had vessel tortuosity<br><br>After HU<br>24/25 (96%) had stable MRIs (Magnetic Resonance Imaging)
**Vasavda et al 2008**	Prospective study In King's College Hospital and St.Thomas Hospital in London	n=30(24 adults and 6 paediatrics)<br><br>Adult and pediatrics Had α-Thalassaemia genotype	Hydroxyurea<br>**Adults**<br>Started at 500mg/d or 15mg/kg/d whichever is higher.<br>Increased in 500mg steps after 4-6wks to MTD<br>**Pediatrics**<br>Started at 15mg/kg/d only increased by 5mg/kg/d.<br>Mean dose(1g/d)<br>300mg-2g.	_	Magnitude change in each parameter because of HU Therapy and this change among SCD patients with co-existing α-thalassaemia (n=10) and those without (n=20).<br>Magnitude change differed significantly for the following parameters;<br>Total Hb p=0.033<br>HbF p=0.024<br>MCV p=0.002<br>MCH p=0.043<br>RBC p=0.035
**Zimmerman et al 2007**	Prospective single institution study at Duke's University Medical centre	n=59,Children with SCA	Hydroxyurea<br>MTD 27.9±2.7mg/kg<br>Median 28.6(18.8-32.6)<br>10±5months of therapy	_	Significant decreases were observed in the right middle cerebral artery (MCA)<br>(166 ± 27cm/s to 135 ± 27cm/s, p<0.001) and left (MCA)(168 ± 26cm/s to 142 ± 27cm/s, p<0.001) velocities<br>The magnitude of the decline in TCD flow velocity was significantly correlated to the maximal baseline TCD flow velocity (r^2=0.12, p=0.04)

**Italia et al 2008**	Prospective cohort study in India.	n=77 patients(48males, 29 females)<br>-Grp 1:29 Adult Sickle homozygous(18-35yrs)<br>-Grp 2:25 Pediatric homozygous(5-17yrs)<br>-Grp 3:23 adult sickle β-Thalassaemia cases(18-35yrs)<br>-Control grp of 20 adults with homozygous SCD.	Hydroxyurea (10-15mg/kg/d)	_	**Mean clinical scores before and After HU therapy**<br>**Before** **After**<br>Grp 1 12.6 ± 1.8 7.2 ± 0.9<br>Grp 2 14.0 ± 1.7 7.5 ± 0.9<br>Grp 3 12.9 ± 1.2 7.3 ± 0.7<br>($p<0.001$)<br><br>**No significant change in the hematological or clinical data was observed among the control group**<br>Clinical score was 11.7±1.2 before and 12.1±1.4 after two years<br>HbF (16.3±7.3% initially and 15.7±6.5% after two years.<br>Mean hemoglobin level was 8.8±0.9g/dl and 8.6±1.1g/dl
**Candrilli et al 2011**	Retrospective longitudinal study in North Carolina Medicaid programme (June 2000 through August 2008)	n=312 met inclusion criteria (mean age 21(±12.2) years.<br>**Inclusion criteria**<br>Medicaid enrollees with SCD	Hydroxyurea	_	35% adherent defined as a medication possession ratio(MPR)≥0.80<br>Mean MPR was 0.60<br>In the twelve months following HU initiation, adherence was associated with:<br>1. Decreased risk of SCD related hospitalization(hazard ratio [HR]=0.65, p=0.0351<br>2. All-cause and SCD related emergency department visit (HR=0.72, p=0.0388; HR=0.58,p=0.0079

		<65yrs.Continuous Medicaid enrollment≥12mnths before and following HU initiation.<br>≥2HU prescriptions.			respectively)<br>3. Vaso- occlusive event (HR=0.66, p=0.0130)<br>4. Adherence was associated with reductions in health care costs such as all-cause and SCD related inpatient(-$5,286,p<0.0001;-$4,403,p<0.0001 respectively) total costs (-$6,529, p<0.0001;-$5,329; p<0.0001 respectively)
**Nzouakou R et al 2010**	Retrospective cohort study in Henri-Mondor Hospital in Creteil and Tenon Hospital in Paris	n= 123 of which n=12 were less than 18years old<br>**Inclusion criteria;** Homozygous SCD patients treated with HU	Hydroxyurea	_	**HU tolerance and safety**<br>41 (33%) patients experienced 66 adverse events, including 4 deaths during a median follow up of 2.8years (range 0.02-10.5; frequency 12% per patient year). The four patients who died during follow up, had all stopped taking HU 1-5 years before their deaths. Causes of death were: toxic shock, severe VOC, heart failure in a patient suffering from pulmonary hypertension and non specified cardiac failure.<br><br>**HU efficacy**<br>The total number of crises during the year preceding HU treatment was 276 (mean 2.8±1.8 per patient-year) which is significantly higher than the 88 crises (mean 0.7 ±0.8 per patient-year) observed during the first year under HU (p<0.0001)<br>For 64 patients, data on hospitalization durations during the year preceding HU were available: a mean decrease of 13.4 days of hospitalization under HU (p<0.0001) was observed
**Ali SB et al 2011**	Prospective cohort study in Sickle cell Unit	N=43(children <18 years of age)	Hydroxyurea(HU)<br>N=10	-No HU n=33	Average HU dose at MTD was 25.4 ± 3.4 mg/kg/day.<br>43 children followed up for 111 person years.

	in West Of Indies in Jamaica	Included children with first clinical stroke between January 1,2000 and September 30, 2009			Of the 10 who agreed to start HU, only one child had clinical stroke recurrence incidence rate 2/100 person years compared to 20/33 in the non-HU group, incidence rate 29/100 person years(Hazard Ratio(HR) 9.4,95% confidence interval 1.3-70.6,p<0.03).<br>In Non-HU group four died versus zero in the HU group.<br>When the Non-HU group was compared to HU group the following was observed:<br>13(53% versus10%) had moderate –severe physical disability (p=0.017) and 12(44% versus 20%) required special education or were too disabled to attend school.
**Mellouli F et al 2007**	Prospective, single centre study of a cohort of clinical patients<br><br>Total follow up time 6yrs 9months<br>Central Hospital, Tunis, Tunisia	27 children with HbSS and 20 children with HbS/BetaThal: 30 male 17 female. Median age at entry 12.5yrs. Excluded children with liver or renal impairment and children with HIV or Hepatitis B	Started on 10-15mg/kg/day but due to formulation to get this dose administration was 3-7 days per week; dose increased to max of 30-35mg/kg/day as tolerated and depending on haematological monitoring	Before and after study within individual patients but 'before data' based on retrospective review of records, then active 2 monthly follow up	Noted 1 episode of severe thrombocytopenia with severe leucopenia that resolved after stopping HU, another episode of pancytopenia was attributed to parvovirus infection<br>• Reduction in mean number of days hospitalized from 29,3 days/yr (95% CI 7–84) to 3,2 days/yr (0-15), P<0.01<br>• 21/38 patients treated with HU for recurrent crises (>3/yr) had no further crises<br>• 7 patients treated with HU post an episode of acute chest syndrome had no further episode<br>• 2 patients treated with HU as prophylaxis after a 1st CVA had no repeat CVA<br>• Reduction in number of transfusions / yr from 4 (range 0-12) to 0.2 (range 0-5), p<0.01<br>There were improvements in HbF, and mean Hb and falls in mean WBC

**Ware et al 2012**	Multicentre Single-masked Noninferiority trial<br><br>26 pediatric sickle cell Programmes	n=161children with Sickle Cell Anemia (SCA), previous stroke and ≥18months of transfusions with documented iron overload.	Hydroxyurea plus overlap transfusions during dose escalation to maximum tolerated dose(MTD) 26.2 ±4.9 mg/kg/d with monthly phlebotomy<br>67 children included in Intention to treat analysis<br>24 completed treatment phase<br>64 completed the 6 month follow up study<br>7 had recurrent stroke	Transfusion and chelation with deferasirox<br>66 children included in intention to treat analysis<br>26 completed treatment phase<br>61 completed the 6 month follow up study<br>1 died on study	**Primary endpoints**<br>**1.Secondary stroke recurrence**<br>• Intervention 7 strokes in 67 (10% had secondary stroke) i.e. 5.6 events per 100 patient years<br>• Comparator 0 strokes in 66 subjects had secondary strokes i.e. 0 events per 100 patient years<br>**2.Iron overload (Liver iron Concentration(LIC) defined as ≥5mg/g dry weight liver**<br>• Intervention 15.7mg/g dry weight liver<br>• Comparator 16.6mg/g dry weight liver<br>After interim analysis the study was closed due to futility in achieving the composite primary endpoint i.e. without reaching superior iron unloading with intervention treatment the observed unbalanced recurrence rates did not warrant study continuation.
